# A novel method for crystalline silicon solar cells with low contact resistance and antireflection coating by an oxidized Mg layer

**DOI:** 10.1186/1556-276X-7-32

**Published:** 2012-01-05

**Authors:** Jonghwan Lee, Youn-Jung Lee, Minkyu Ju, Kyungyul Ryu, Bonggi Kim, Junsin Yi

**Affiliations:** 1 School of Information and Communication Engineering, Sungkyunkwan University, Suwon, 440-746, South Korea; 2 Department of Energy Science, Sungkyunkwan University, Suwon, 440-746, South Korea

**Keywords:** solar cell, Mg metal film, low-series contact resistance, antireflection coating.

## Abstract

One of the key issues in the solar industry is lowering dopant concentration of emitter for high-efficiency crystalline solar cells. However, it is well known that a low surface concentration of dopants results in poor contact formation between the front Ag electrode and the n-layer of Si. In this paper, an evaporated Mg layer is used to reduce series resistance of c-Si solar cells. A layer of Mg metal is deposited on a lightly doped n-type Si emitter by evaporation. Ag electrode is screen printed to collect the generated electrons. Small work function difference between Mg and n-type silicon reduces the contact resistance. During a co-firing process, Mg is oxidized, and the oxidized layer serves as an antireflection layer. The measurement of an Ag/Mg/n-Si solar cell shows that *V*_oc_, *J*_sc_, FF, and efficiency are 602 mV, 36.9 mA/cm^2^, 80.1%, and 17.75%, respectively. It can be applied to the manufacturing of low-cost, simple, and high-efficiency solar cells.

## Background

The main objective of the current single crystalline silicon [c-Si] photovoltaic research is to enhance the efficiency of the solar cell without a too complicated process. One of the ways to increase the efficiency is to lower the front surface recombination using a low surface doping concentration. C-Si solar cells with a lowly doped emitter have high short-circuit current, and the absorption in the blue response region is better. It has been reported that the recombination velocity of the emitter layer with a sheet resistance of 100 Ω/sq is 60,000 cm/s while that of the emitter with a 45-Ω/sq sheet resistance is 180,000 cm/s [1].

The quality of metal contact can also affect the solar cell efficiency. For commercial c-Si solar cells, the front contact metallization is fabricated by the screen printing of Ag paste in grid patterns [2,3]. The screen printed Ag electrodes may reduce the efficiency when operated at concentration levels of more than 1 sun due to high-series resistance. A small front metal contact area [4,5] and a low metal density in Ag-screen printed lines contribute to the high resistance [6]. The presence of a glass frit mostly contributed by SiO_2 _in the Ag paste also results in high metal-semiconductor contact resistivity [7]. Increased resistive power loss occurs under light and eventually degrades the solar cell efficiency [8]. To reduce the contact resistance, a selective emitter method is introduced recently. It consists of a lightly doped emitter to enhance the blue response of solar cells and a heavily doped emitter underneath the contact to reduce the contact resistance. Although the performance of the selective emitter solar cell is fairly good, its fabrication process is very complicated, making it difficult for mass production [9]. To have similar effects without using the selective emitter method, a barrier material can be introduced between the Ag and Si since it is difficult to get ideal ohmic contacts with an n-type silicon substrate doped with phosphorus. The contact resistance is decided by the doping density of impurity and the work function difference between Si and the metal. To achieve decreased contact resistance, it is important to have a low work function difference between Si and the metal. One of the good candidates for the barrier is Mg [10]. In this paper, Mg is inserted between Ag and Si. The effects of Mg on the contact resistance and the reflectance are investigated.

## Methods

**Figure 1:**
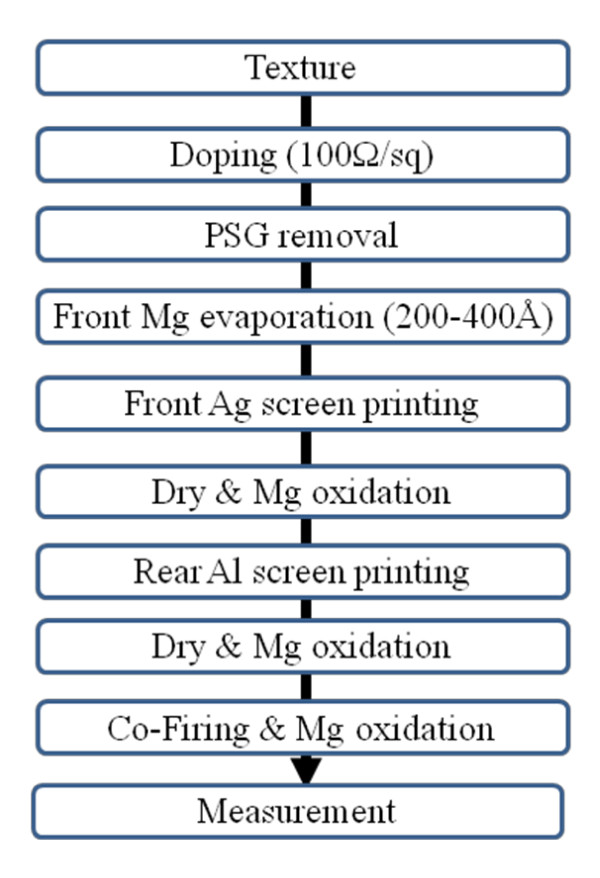
**A flow diagram of the processed solar cells with Mg evaporation and oxidation**.

Figure 1 shows the processing sequence of the solar cell using the Mg metal. The substrates used were Czochralski (Cz) and (100) boron-doped, p-type silicon wafers having a resistivity of 1.5 Ω cm, a thickness of about 200 μm, and a size of 1.5 × 1.5 cm. Initially, the wafers were textured by a 1% NaOH solution followed by an n+ layer formed by POCl_3 _doping. Doping was carried out at 810°C for 7 min, and the resulting sheet resistance was 100 Ω/sq. A phosphorous silicate glass layer was removed and rinsed with a 10% hydrofluoric acid [HF] solution followed by de-ionized [DI] water. The wafers were then dipped in the HF solution for 30 s followed by DI water rinsing and drying. Thin Mg layers having thicknesses of 200, 300, and 400 Å were deposited using a thermal evaporator. The thicknesses of Mg layers were monitored by alpha-step. The amount of Mg used was easily controlled using Mg pellets that weighed 0.1 g each. The front electrode was screen printed with a conventional Ag paste; the rear side was printed with an Al paste. The front and rear electrodes were dried with an infrared [IR] belt furnace at 150°C. During the drying processes, the Mg layer was oxidized. After the first oxidation, the lifetime was measured with a Sinton WCT-120 (Boulder, CO, USA) by quasi-steady-state photoconductance decay [11]. The reflectance was measured using a Sinton S-3100 UV-visible spectrometer. Finally, co-firing of the front-side and backside electrodes was done in a four-zone IR belt furnace with peak temperatures ranging from 725°C to 850°C. The region where Ag is in contact with Mg formed an electrode having very low contact resistance. The rest of the wafer was covered with oxidized Mg acting as antireflection coating [ARC] The illuminated current-voltage characteristics under the global solar spectrum of AM 1.5 at 25°C, dark current-voltage [DIV] characteristics, and the internal quantum efficiency [IQE] of each cell were studied.

## Results and discussion

The cross section through the contact shows that the screen printed Ag electrode is in contact with the pure Mg metal layer on Si, resulting in low contact resistance. The rest of the Mg part undergoes an oxidation process becoming MgO, acting as an ARC layer. The passivation capabilities of MgO layers formed under various experimental conditions are investigated.

**Figure 2:**
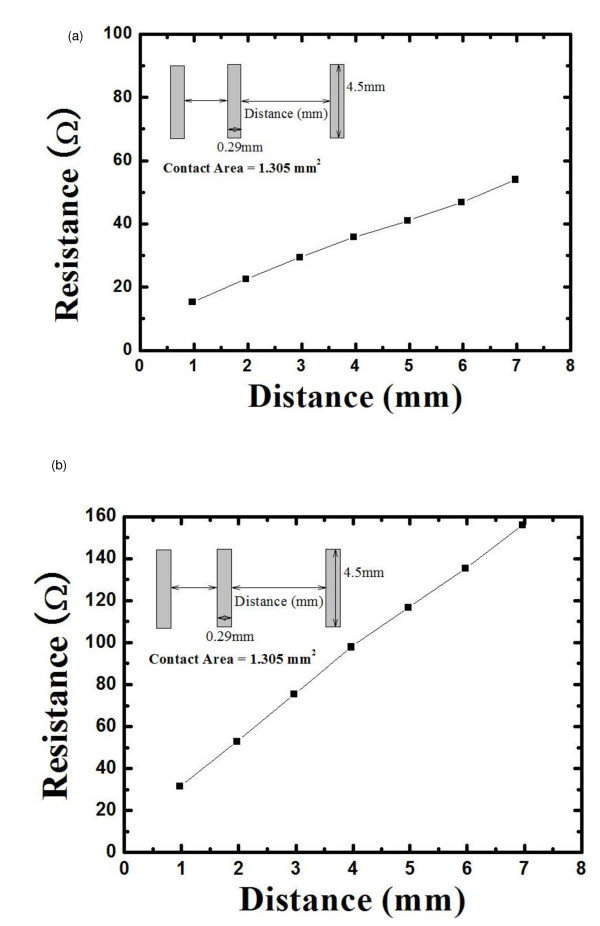
**TLM results of (a) the Mg/n-Si contact resistance and (b) the Ag/n-Si contact resistance**.

In the case of the lightly doped silicon (*N*_D _< 17 cm^-3^), the current flow at the interface is mostly decided by thermion emission, while field emission is the main mechanism in the case of the heavily doped silicon (*N*_D _> 17 cm^-3^). In both cases, the most important factor that affects the current flow is the work function difference between Si and metal. A lightly doped emitter results in increased contact resistance between contact finger and n-Si. Since the screen printed Ag metal paste forms too high potential barrier to form an ohmic contact with an n+ region, a new material can be inserted between Ag and Si to form low barrier height (Φ_B_) and thus to reduce the contact resistance. Among various materials, Mg metal is selected to lower the contact resistance. The transfer length method [TLM] is commonly used to model the planar metal-semiconductor contact, allowing important contact parameters such as contact resistance *R*_c _(in ohms), specific contact resistivity *ρ*_c _(in ohm centimeter squared), and the semiconductor sheet resistance beneath the contact *R*_s _(in ohm centimeters). The TLM consists of sets of resistors representing the metal, diffusion, and interfacial layers of a contact [12]. The contact resistance between Mg and n-Si can be extrapolated by the following formula:

(1) $$R_{\text{T}}=\rho_{\text{c}}d∕z+\text{2}R_{\text{c}},$$

where *R*_T _is the total resistance, and *R*_c _is the contact resistance. The total resistance is measured for various contact spacings; *R*_c _and *R*_T _are plotted as functions of *d*. The intercept at *d *= 0 is *R*_T _= 2*R*_c_, giving the contact resistance. To calculate the contact resistance, Mg was evaporated on the n-Si wafer, and the Ag paste was printed on the Si wafer for reference. The contact resistances of Mg/Si and Ag/Si were measured by the TLM method (Figure 2). The contact resistivity of the Mg/Si system was characterized to be 9.535 × 10^-3 ^Ω cm^2 ^which is even lower than that of Ag/Si, 8.64 × 10^-^2 Ω cm^2^. It was found that Mg/Si was suitable for a high-efficiency solar cell application.

**Figure 3:**
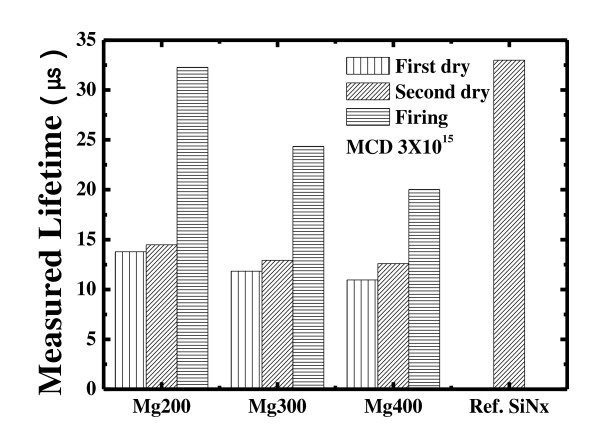
**Carrier lifetimes of Si wafers with different Mg thicknesses and sintering steps**. Mg200, thickness of Mg is 200 Å; Mg300, thickness of Mg is 300 Å; Mg400, thickness of Mg is 400 Å.

Figure 3 shows the carrier lifetimes of Si wafers with different Mg thicknesses (200 to 400 Å) and sintering steps. After the first oxidation, the lifetimes of Mg200, Mg300, and Mg400 were 13.78, 11.84, and 10.9 μs, respectively. The second oxidation does not affect the lifetime much, showing that the oxidation process at 150°C does not contribute to the oxidization of Mg. However, a sharp increase in the lifetime is seen after the last oxidation especially in the case of Mg200. The lifetimes of Mg200, Mg300, and Mg400 increase from 13.79, 11.84, and 10.95 μs to 32.27, 24.35, and 20.03 μs, respectively. When the deposited layer of the Mg metal is thin, the thickness of MgO formed after the oxidation process is also thin and does not serve as a good passivation or ARC layer. It shows that the best passivation is obtained when the thickness of the Mg metal is around 200 Å. As the amount of Mg increases, the resistance increases and the passivation deteriorates. From the results, a 200-Å thickness of Mg is required to form a desired dense MgO layer [13].

**Figure 4:**
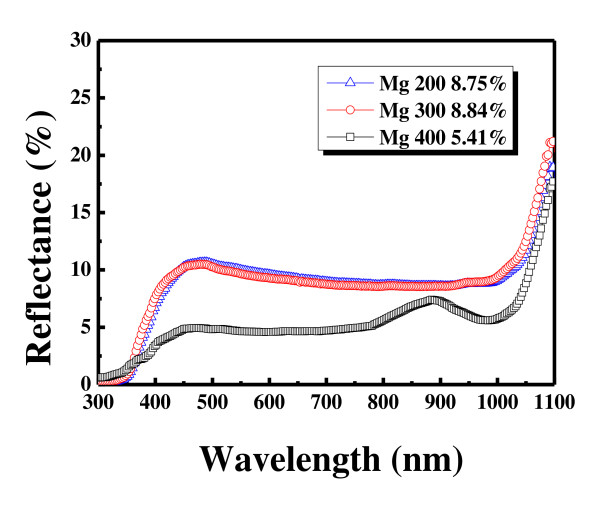
**Dependence of reflectance on the Mg thickness and oxidation steps**.

**Figure 5:**
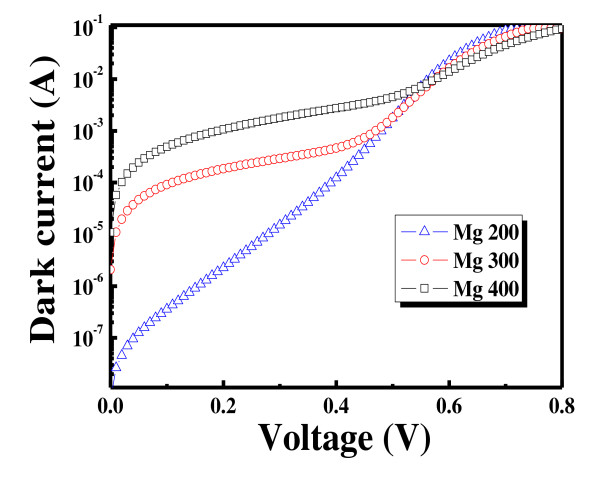
**Effect of Mg thickness on the DIV of the processed solar cells**.

**Figure 6:**
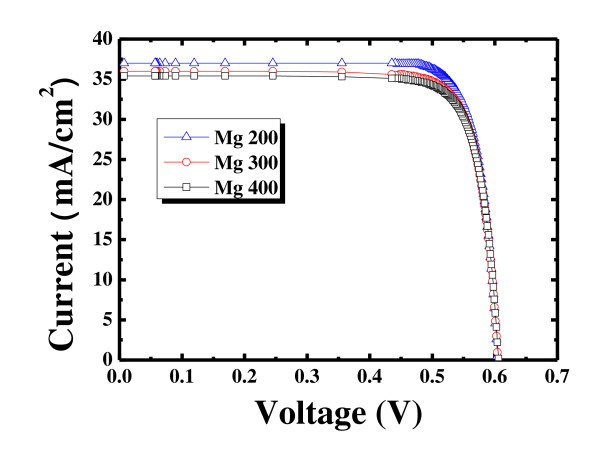
**Sun-*V*_oc _data of the processed solar cells with different Mg thicknesses**.

Figure 4 shows the dependence of reflectance on Mg thickness and oxidation steps. Before the oxidation, the reflectance of the Mg metal increases as the thickness increases. The solar cell current cannot be measured because of the high reflectance. To solve this problem, Mg is oxidized and MgO is formed. The reflectance of Mg is decreased dramatically after the oxidation. The reflectances of Mg200, Mg300, and Mg400 decrease from 25%, 31.62%, and 47.05% to 8.75%, 7.84% and 5.41%, respectively. This shows that the optimum thickness of the Mg metal which minimizes the reflectance at this wavelength region has been deposited successfully. The optimized thickness of the Mg metal layer is extracted from the following formula:

(2) $$n_{\text{1}}\times d=\frac{\lambda_{0}}{4},$$

where *λ*_0 _is 550 nm (where the strongest intensity of sunlight is emitted), *n*_1 _is the refractive index of MgO (which is equal to 1.74), and *d *is the thickness of the ARC layer. Thicker MgO results in less reflectance as its thickness gets close to 79 nm which is reported to be an optimized thickness of MgO for the ARC layer of solar cells [13]. Figure 5 and Table 1 show the DIV measured. The current is measured in the potential range of 0 to 0.8 V. The current of the PN junction diode can be separated into two regions: the quasi-neutral region (*n*_1_) and the space-charge region (*n*_2_) recombination/generation [14]. In the two-diode model, this deviation is taken into account by including a second diode. The second diode expresses generation and recombination currents within the space-charge region. When the ideality factor of the second diode deviates far from 2, leakage current increases, parallel resistance [*R*_sh_] decreases, and series resistance [*R*_s_] increases. Increased leakage current and decreased *R*_sh _cause a decrease in the short-circuit current density [*J*_sc_]. Increased *R*_s _and decreased *R*_sh _result in a sharp decline in the fill factor [FF]. In Table 1, it is shown that the leakage current gets higher as the thickness of the Mg metal increases. This is due to the fact that a thicker Mg metal digs more into the Si substrate and results in poor solar cell performance. The extracted optimized conditions of the Mg metal layer are applied to the c-Si solar cell, and its properties are measured. Figure 6 and Table 2 show the sun-open-circuit voltage [*V*_oc_] characteristics of solar cells with different Mg thicknesses. It is seen that the cell with Mg200 Å deposition has the best efficiency. The *V*_oc _of the cell is 602 mV, and the *J*_sc _is 36.9 mA/cm^2^. Relatively low *V*_oc _value is obtained, and it is attributed to the reduced band bending caused by a low doping concentration of the emitter layer. However, the *J*_sc _value is relatively high due to the effect of the high *R*_s _emitter and the MgO layer collecting more carriers which would have usually been recombined at the front surface. Consequently, with this high *J*_sc _value, the high-*R*_s _c-Si solar cell reaches a conversion efficiency of 17.75%.

**Table 1:** Series resistance, shunt resistance, first ideality factor, and second ideality factor of processed solar cells

	Mg200 Å	Mg300 Å	Mg400 Å
*R*_s _(Ω cm)	1.04	1.36	1.65
*R*_sh _(Ω)	24,948.2	984.8	182.8
*n*_1 _	1.38	1.67	4.08
*n*_2 _	2.03	9.02	8.01
*J*_oe _(A/cm^2^)	5.22E-09	6.44E-08	1.51E-04

*R*_s_, series resistance; *R*_sh_, parallel resistance; *n*_1_, quasi-neutral region; *n*_2_, space-charge region; *J*_oe_, saturation current density.

**Table 2:** Sun-*V*_oc _parameters of the processed solar cells with different Mg thicknesses without an oxidation step

Sample	***V***_**oc**_ (mV)	***J***_**sc**_ (mA/cm^2^)	FF (%)	EFF (%)
Mg200 Å	602	36.9	80.1	17.75
Mg300 Å	598	36	78.6	16.99
Mg400 Å	600	35	78.4	16.67

*V*_oc_, open-circuit voltage; *J*_sc_, short-circuit current density; FF, fill factor; EFF, efficiency.

Generally, the quantum efficiency is reduced by recombination. The same mechanisms which affect the collection probability also affect the quantum efficiency. Since blue light is absorbed very close to the surface, high front surface recombination will affect the blue portion of the quantum efficiency. Thus, a good front surface passivation is important. Green light is absorbed in the bulk of a solar cell, and a low diffusion length reduces the quantum efficiency in the green portion of the spectrum. The quantum efficiency can be viewed as the collection probability due to the generation profile of a single wavelength, integrated over the device thickness and normalized to the incident number of photons. From the IQE graph, not presented here, from a wavelength of *λ = *400 to 1,100 nm, the best blue response is seen for the cell with a 200-Å-thick Mg layer, suggesting that it has the most decreased recombination velocity at the surface.

## Conclusion

In conventional solar cells, a screen printed Ag paste is often used for front metallization materials. It induces the increase of contact resistance due to high barrier height between Ag and n-type Si. To solve this problem, Mg metal is evaporated before the formation of Ag electrode. After the Ag electrode is screen printed, a firing step is taken. As a result, a low contact resistance is obtained at the Ag/Mg/Si electrode. Since the exposed Mg metal goes through oxidation during firing, it becomes MgO serving as an ARC layer. Since the whole emitter is lightly doped (100 Ω/sq), the surface recombination velocity is reduced by the simple and low-cost fabrication process.

Optimum thickness of Mg has been investigated. It is found that the lifetime is 32.27 μs and the reflectance is 8.75% when the Mg thickness is 200 Å. This condition is applied to a 100-Ω/sq c-Si solar cell process to have low contact resistance with good ARC. The measurement of an Ag/Mg/n-Si solar cell shows that *V*_oc_, *J*_sc_, FF, and efficiency are 602 mV, 36.9 mA/cm^2^, 80.1%, and 17.75%, respectively.

## Competing interests

The authors declare that they have no competing interests.

## Authors' contributions

JL proposed the original idea, carried out the synthesis and analysis, and wrote the first draft of the manuscript. MJ carried out most of the experiments with JL and shared his idea with the other authors. KR, Y-JL, and BK detailed the original idea and modified the first draft of the manuscript. JY designed and coordinated the whole work and finalized the manuscript. All authors read and approved the final manuscript
